# The utility of the brain trauma evidence to inform paramedic rapid sequence intubation in out-of-hospital stroke

**DOI:** 10.1186/s12873-020-0303-9

**Published:** 2020-01-28

**Authors:** Pieter Francsois Fouche, Paul Andrew Jennings, Malcolm Boyle, Stephen Bernard, Karen Smith

**Affiliations:** 1 Department of Community Emergency Health and Paramedic Practice, Monash University, Melbourne, Australia; 2 Ambulance Victoria, Doncaster, Australia; 3 School of Medicine, Griffith University, Gold Coast, Australia; 4 Research and Evaluation, Ambulance Victoria, Blackburn North, Australia; 5 Department of Epidemiology and Preventive Medicine, Monash University, Melbourne, Australia

**Keywords:** Traumatic brain injury, Stroke, rapid sequence intubation, Paramedic

## Abstract

**Background:**

Rapid sequence intubation (RSI) is used to secure the airway of stroke patients. Randomized controlled trial evidence exists to support the use of paramedic RSI for traumatic brain injury (TBI), but cannot necessarily be applied to stroke RSI because of differences between the stroke and TBI patient. To understand if the TBI evidence can be used for stroke RSI, we analysed a retrospective cohort of TBI and strokes to compare how survival is impacted differently by RSI when comparing strokes and TBI.

**Methods:**

This study was a retrospective analysis of 10 years of in-hospital and out-of-hospital data for all stroke and TBI patients attended by Ambulance Victoria, Australia. Logistic regression predicted the survival for ischemic and haemorrhagic strokes as well as TBI. The constituents of RSI, such a medications, intubation success and time intervals were analysed against survival using interactions to asses if RSI impacts survival differently for strokes compared to TBI.

**Results:**

This analysis found significant interactions in the RSI-only group for age, number of intubation attempts, atropine, fentanyl, pulse rate and perhaps scene time and time- to-RSI. Such interactions imply that RSI impact survival differently for TBI versus strokes. Additionally, no significant difference in survival for TBI was found, with a − 0.7% lesser survival for RSI compared to no-RSI; OR 0.86 (95% CI 0.67 to 1.11; *p* = 0.25). Survival for haemorrhagic stroke was − 14.1% less for RSI versus no-RSI; OR 0.44 (95% CI 0.33 to 0.58; *p* = 0.01) and was − 4.3%; OR 0.67 (95% CI 0.49 to 0.91; *p* = 0.01) lesser for ischemic strokes.

**Conclusions:**

Rapid sequence intubation and related factors interact with stroke and TBI, which suggests that RSI effects stroke survival in a different way from TBI. If RSI impact survival differently for strokes compared to TBI, then perhaps the TBI evidence cannot be used for stroke RSI.

## Background

Strokes account for 10% of deaths worldwide [1]. Rapid Sequence intubation (RSI) is used in the emergency setting to improve survival in strokes, with perhaps 6 to 79% of strokes receiving intubation, depending on the stroke type [2]. Rapid sequence intubation is used to secure the airway using sedative and paralytic drugs to facilitate endotracheal intubation [2]. Strokes form 37% of RSI for non-traumatic brain pathologies undertaken by paramedics in Victoria, Australia [3]. Despite the not-infrequent use of RSI in unconscious out-of-hospital acquired brain injuries, no high quality evidence exists to support the use of RSI for strokes [2–4]. A randomized controlled trial of RSI in traumatic brain injury (TBI) exist [5], but the evidence from this trial cannot necessarily be applied to stroke RSI due to differences between the stroke and TBI patient [2–4].

Lower RSI survival for strokes compared to TBI suggests that the evidence from TBI might not be applicable to RSI in stroke [4]. It is important to investigate if brain trauma RSI evidence is transferable to strokes. That is, it would be vital to understand the RSI components that cause the survival difference of TBI compared to strokes. This study aims to find the constituents of RSI that could cause different survival between TBI and strokes. If any component of RSI impacts survival differently for TBI compared to strokes, it would follow that RSI itself has a different effect on these two pathologies. If RSI causes dissimilar survival for these two illnesses, then this would mean that the RSI TBI evidence-base cannot be transferred to strokes. Any such differences would imply that a stroke RSI trial is needed.

## Methods

### Study setting and data sources

Victoria has 6.3 million residents serviced by a two-tier emergency medical service. Mobile Intensive Care Paramedics are authorized to provide RSI to patients that have a Glasgow Coma Scale of less than 10 using suxamethonium as the primary paralytic, with pancuronium used to maintain paralysis [6]. Midazolam, morphine/midazolam infusions, atropine, ketamine and fentanyl were available to aid RSI [6]. This study analysed data from 131 hospitals and clinics in Victoria, Australia for the 10 year period 1 January 2008 to 31 December 2017. Monash University Human Research Ethics Committee provided ethics approval (ref. no. 8618).

### Selection of cohort

This study included all patients of any age that were treated and transported by Ambulance Victoria, with a hospital diagnosis of TBI or stroke. We excluded transient ischemic attack and strokes that could not be classified as either haemorrhagic or ischemic. Instances of stroke and TBI were identified by the Australian modification of ICD10 codes: S06 (Intracranial injury), I60 (subarachnoid haemorrhage), I61 (intracerebral haemorrhage), I62.9 (intracranial haemorrhage [non-traumatic], unspecified) and I63 (cerebral infarction). We initially selected all hospital records of patients with TBI and stroke codes, irrespective of ambulance transportation. These TBI and stroke in-hospital records were then linked to the Ambulance Victoria out-of-hospital records to select those that had a stroke or TBI and were transported by ambulance [4].

### Predictors and outcomes

The primary outcome was survival to hospital discharge. Potential predictors include demographic, treatment, baseline observations (“vital signs”) and scene/transport time intervals. Illness severity and comorbidity are important confounders in strokes and TBI, and we adjusted for illness severity using Glasgow Coma Scale. Whilst Glasgow Coma Scale was not specifically designed as a severity score for TBI and strokes, it could be similarly predictive of in-hospital survival as NIHSS for strokes [7], and has strong prognostic value in TBI [8]. We adjusted for comorbidity by calculating the Walraven-Elixhauser comorbidity score [9]. No adjustment for any in-hospital interventions were done to avoid conditioning on a mediator variable [10].

### Definitions

Strokes and TBI are defined by ICD10-AM codes and subtyped for ischemic and haemorrhagic strokes as well as TBI. Rapid sequence intubation is defined as the attempted or successful placement of an endotracheal tube in the trachea after receiving a paralytic agent. Successful placement of endotracheal tube in the trachea was confirmed using clinical means and end-tidal CO_2_ waveform.

### Statistical analysis

Stata version 14 (Stata Corp, College Station, Texas, USA) was used to analyse data. Categorical variables are presented as frequencies, and continuous variables as means with standard deviations. Categorical variables were compared with the χ^2^ test and continuous predictors with the t-test or Wilcoxon rank-sum test. Hypothesis tests were two-sided, with a significance level of *p* < 0.05.

### Model building

We utilized a maximum likelihood-fitted logistic regression model on complete observations only. Non-linear main-effect terms were fitted using fractional polynomials. Goodness of fit was tested using the Hosmer and Lemeshow test on a random sample, due to the tests excess sensitivity to large datasets [11, 12]. Four regression models were assembled. The first was built on all non-missing observations of TBI and strokes to test interactions. Three additional models were constructed to calculate the effect of RSI for TBI, ischemic and haemorrhagic stroke separately. Previous work using this same dataset showed that missingness were clinically insignificant and did not meaningfully impact estimates [4]. As such, no sensitivity analyses for the impact of missing data were conducted.

### Interaction

This analysis tested how the components of RSI interact with stokes and TBI for survival in the RSI-only group. Any such interactions in the RSI-only group might indicate that RSI influences survival differently for strokes compared to TBI, suggesting that the brain trauma RSI evidence cannot be applied to strokes. Rapid sequence intubation consists of many elements including medications, intubation success proportions and number of attempts, scene time and time to intubation. All these factors were tested for interaction and all interactions are presented graphically in this text.

Medications assessed for interaction with TBI and strokes include atropine, midazolam, midazolam/morphine infusions and fentanyl, as only these medications had sufficient data permitted an interaction test. Furthermore, RSI is likely to directly impact blood pressure, pulse rate, oxygen saturation, Glasgow Coma Scale, respiratory rate, end-tidal carbon dioxide [4]. Therefore, we also tested the interactions of vital signs. These vital sign changes were calculated by subtracting the final on-scene value from the first on-scene measurement [4]. All interaction terms that were statistically significant at a 5 % level were considered as evidence of interaction. Results of interactions are presented in the manner suggested by Knol and van Der Weele [13], and we present the relative excess risk due to interaction in supplementary tables [14].

## Results

**Fig. 1:**
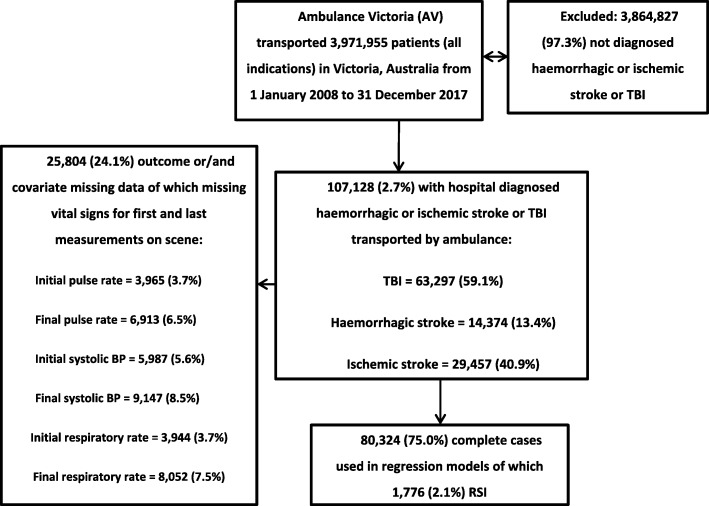
Patient selection for a cohort of RSI in stroke and TBI

This cohort included 107,128 (Fig. 1) of which 1993 (1.9%) received RSI. A total of 80,324 (75%) had complete data of which 11,133 (13.9%) were diagnosed with haemorrhagic stroke and 26,754 (33.3%) with ischemic stroke and 42,437 (52.8%) with TBI. Baseline characteristics of the full-data cohort are compared in Table 1. The overall intubation success was 97.8% and first pass success was 89.6%, with no significant change in success over time for overall (*p* = 0.40) or first-pass (*p* = 0.12).

**Table 1:** Patient, demographic, prognostic and in-hospital factors in in a cohort of 80,324 strokes and traumatic brain injury

Characteristic	Patients, No. (%)		
	Total (*n* = 80,324)	RSI (*n* = 1776)	No-RSI (*n* = 78,548)
Demographic			
Age, mean (SD), years	61.6 (22.5)	52.2 (22.5)	61.8 (24.2)
Sex			
Male	46,726 (58.1)	1125 (63.3)	45,601 (58.1)
Female	33,598 (41.8)	651 (36.7)	32,947 (41.6)
Illness			
Haemorrhagic stroke	11,133 (13.9)	531 (29.9)	10,602 (13.5)
Ischemic stroke	26,754 (33.3)	242 (13.6)	26,512 (33.8)
Traumatic brain injury	42,437 (52.9)	1003 (56.5)	41,434 (52.8)
Illness severity/comorbidity, mean (SD)			
Elixhauser comorbidity score^a^	17.2 (7.0)	17.3 (6.6)	17.2 (7.0)
Initial Glasgow Coma Scale	13.5 (2.8)	6.4 (3.6)	13.7 (2.5)
Observations, mean (SD)			
Initial pulse rate	85.4 (19.5)	92.9 (28.8)	85.2 (19.2)
Final pulse rate	82.6 (18.0)	101.5 (22.4)	82.1 (17.6)
Initial systolic blood pressure	140.6 (31.5)	145.7 (41.8)	140.5 (31.2)
Final systolic blood pressure	139.3 (28.5)	136.4 (29.7)	139.3 (28.5)
Initial respiratory rate	17.7 (4.6)	18.3 (8.0)	17.7 (4.5)
Final respiratory rate	16.9 (4.0)	12.2 (7.5)	17.0 (3.8)
Initial SPO_2_	96.1 (5.1)	94.1 (9.6)	96.1 (4.9)
Final SPO_2_	97.2 (3.4)	98.5 (4.6)	97.2 (3.3)
Ambulance time intervals, minutes mean (SD)			
Response time	21.1 (23.1)	19.2 (18.9)	21.2 (23.2)
Scene time	24.3 (99.9)	60.7 (35.0)	23.4 (100.7)
Transport time	25.6 (21.0)	31.5 (23.5)	25.5 (20.9)
Hospital			
Time in intensive care unit, mean (SD), hours	125.2 (161.3)	156.9 (172.2)	119.4 (158.5)
Mechanical ventilation in intensive care unit, mean (SD), hours	106.8 (148.9)	117.9 (144.6)	103.8 (150.0)
Hospital length of stay, mean (SD), days	6.8 (10.2)	13.3 (20.5)	6.6 (9.8)
Emergency department length of stay mean (SD), minutes	426.1 (318.7)	272.2 (214.8)	430.2 (320.0)
Year			
2008	6497 (8.1)	148 (8.3)	6349 (8.1)
2009	7435 (9.3)	142 (8.0)	7293 (9.3)
2010	7834 (9.8)	167 (9.4)	7667 (9.8)
2011	8614 (10.7)	215 (12.1)	8399 (10.7)
2012	8293 (10.3)	205 (11.5)	8088 (10.3)
2013	7962 (9.9)	194 (10.9)	7768 (9.9)
2014	6416 (8.0)	123 (6.9)	6293 (8.0)
2015	8581 (10.7)	164 (9.2)	8417 (10.7)
2016	9101 (11.3)	221 (12.4)	8880 (11.3)
2017	9591 (11.9)	197 (11.1)	9394 (12.0)

^a^Scaled to avoid negative values

### Overall outcomes

After removing missing data, 80,324 strokes and TBI were transported by ambulance of which 71,008 (88.4%) survived to hospital discharge. In an unadjusted regression those that had received RSI had 88% lesser odds of survival; OR 0.09 (95% CI 0.08 to 0.10; *p* < 0.001) compared to those patients that did not receive RSI. An adjusted regression analysis found no significant difference in survival for TBI, with − 0.7% lesser survival for RSI compared to no-RSI; OR 0.86 (95% CI 0.67 to 1.11; *p* = 0.25) (Additional file 1: Table S1A). Survival for haemorrhagic stroke was − 14.1% less for RSI; OR 0.44 (95% CI 0.33 to 0.58; *p* = 0.01) and was − 4.3%; OR 0.67 (95% CI 0.49 to 0.91; *p* = 0.01) lesser for ischemic strokes (Additional file 1: Table S1B and C). Model fit and performance statistics indicate good fit (Additional file 1: Table S3).

Table 2 shows that the decline in systolic blood pressure is tenfold larger for haemorrhagic strokes compared to TBI. Oxygen saturation increases more when RSI is utilized, and the largest rise is for ischemic strokes. Respiratory rate does not change meaningfully in the absence of RSI use, but there are six breaths per minute drop with RSI. Predictably, Glasgow Coma Scale decreases with RSI usage. For non-RSI patients pulse rate tends to decrease slightly on scene, but when RSI is utilized pulse rate increases, with haemorrhagic stroke double that of TBI and ischemic strokes.

**Table 2:** Comparison of changes in vital signs in a cohort of 80,324 strokes and traumatic brain injury

Change in vital sign^a^	RSI (mean; 95% CI)	No-RSI (mean; 95% CI)	Difference (95% CI; P)
Haemorrhagic strokes			
Systolic blood pressure (mmHg)	−15.8 (−17.8 to −13.9)	−2.0 (−2.4 to −1.6)	−13.8 (− 15.8 to − 11.8; *p* < 0.001)
SPO_2_ (%)	2.0 (1.4 to 2.5)	1.8 (1.7 to 2.0)	0.1 (−0.5 to 0.7; *p* = 0.72)
Respiratory rate (per minute)	−5.8 (−6.1 to −5.5)	−0.4 (− 0.5 to − 0.4)	−5.4 (− 6.1 to − 5.5; *p* < 0.001)
Glasgow Coma Scale (unit)	−3.5 (−3.6 to −3.3)	− 0.08(− 0.1 to − 0.04)	−3.4 (− 3.5 to − 3.2; *p* < 0.001)
Pulse (per minute)	13.2 (11.9 to 14.3)	−1.7 (− 1.9 to − 1.4)	14.8 (13.6 to 16.1; *p* < 0.001)
Ischemic strokes			
Systolic blood pressure (mmHg)	−9.8 (− 12.1 to − 1.1)	−1.3 (− 1.5 to − 1.1)	−8.5 (− 10.8 to − 6.2; *p* < 0.001)
SPO_2_ (%)	4.2 (3.6 to 4.8)	1.2 (1.1 to 1.3)	3.0 (2.3 to 3.6; *p* < 0.001)
Respiratory rate (per minute)	−6.8 (−7.1 to − 6.5)	−0.5 (− 0.48 to − 0.43)	− 6.4 (− 6.7 to − 6.1; *p* < 0.001)
Glasgow Coma Scale (unit)	−3.9 (− 4.1 to − 3.8)	0.1 (0.1 to 0.14)	− 4.0 (− 4.2 to − 3.9; *p* < 0.001)
Pulse (per minute)	4.7 (3.3 to 6.0)	−1.5 (− 1.6 to − 1.4)	6.2 (4.8 to 7.6; *p* < 0.001)
Traumatic brain injuries			
Systolic blood pressure (mmHg)	−2.6 (− 3.6 to − 1.5)	−1.3 (− 1.4 to − 1.1)	−1.3 (− 2.4 to − 0.2; *p* = 0.02)
SPO_2_ (%)	2.2 (1.8 to 2.5)	1.0 (0.9 to 1.1)	1.2 (0.8 to 1.5; *p* < 0.001)
Respiratory rate (per minute)	−6.1 (− 6.3 to − 5.9)	−0.9 (− 0.9 to − 0.85)	− 5.2 (− 5.4 to − 5.0; *p* < 0.001)
Glasgow Coma Scale (unit)	−5.6 (− 5.7 to − 5.5)	0.3 (0.2 to 0.3)	−5.8 (− 5.9 to − 5.7; *p* < 0.001)
Pulse (per minute)	7.4 (6.6 to 8.2)	−4.5 (− 4.6 to − 4.4)	11.9 (11.0 to 12.7; *p* < 0.001)

^a^All changes in vital signs calculated as the last minus the first measurement on scene. Means are adjusted for age, initial GCS, year, initial respiratory rate, initial pulse rate, initial systolic blood pressure and Elixhauser comorbidity score

### Interactions

**Fig. 2:**
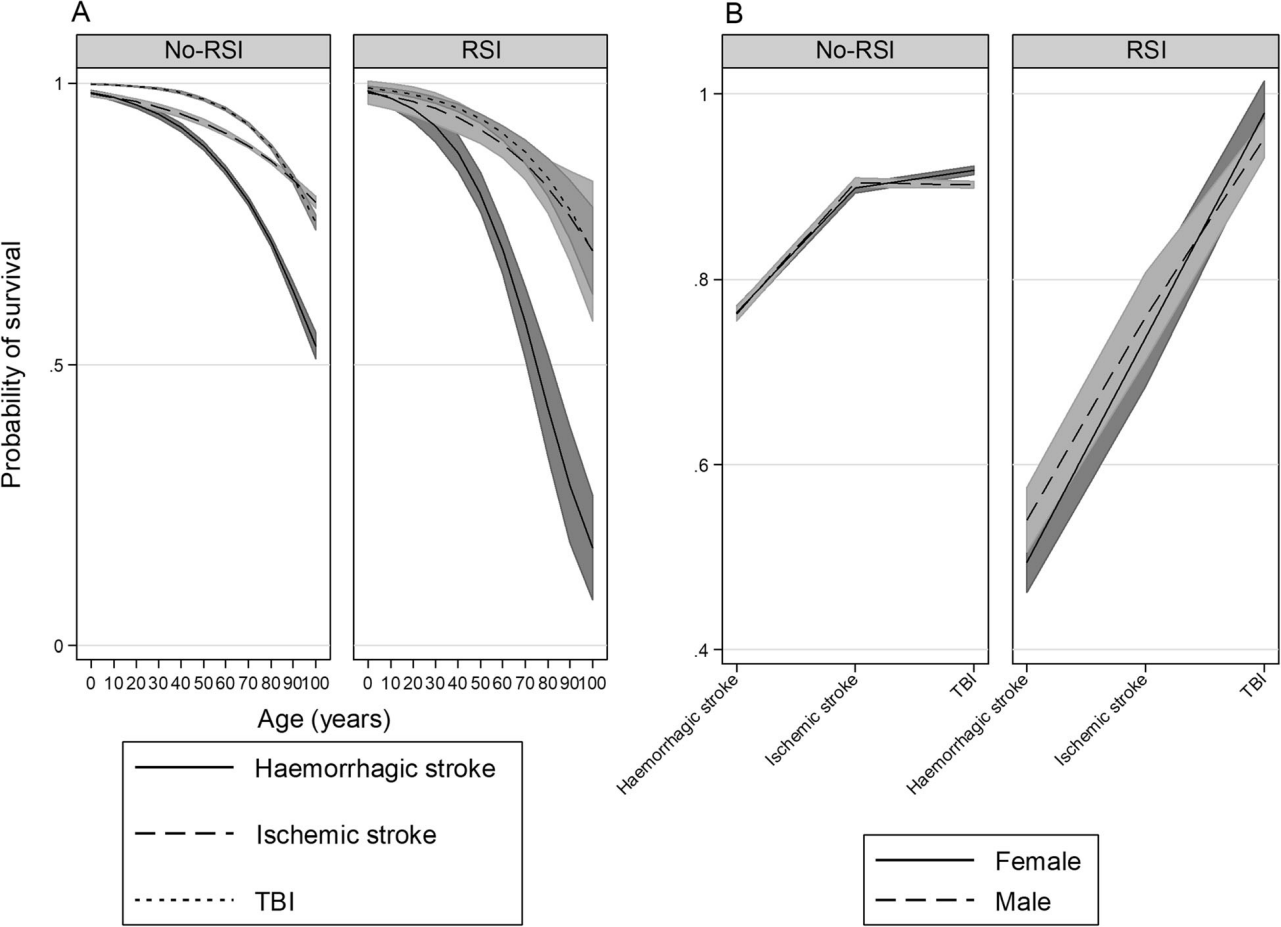
Interactions of demographic factors in a cohort of strokes and TBI, RSI compared to no-RSI. **a** interaction of age and pathology. **b** interaction of sex and pathology

**Fig. 3:**
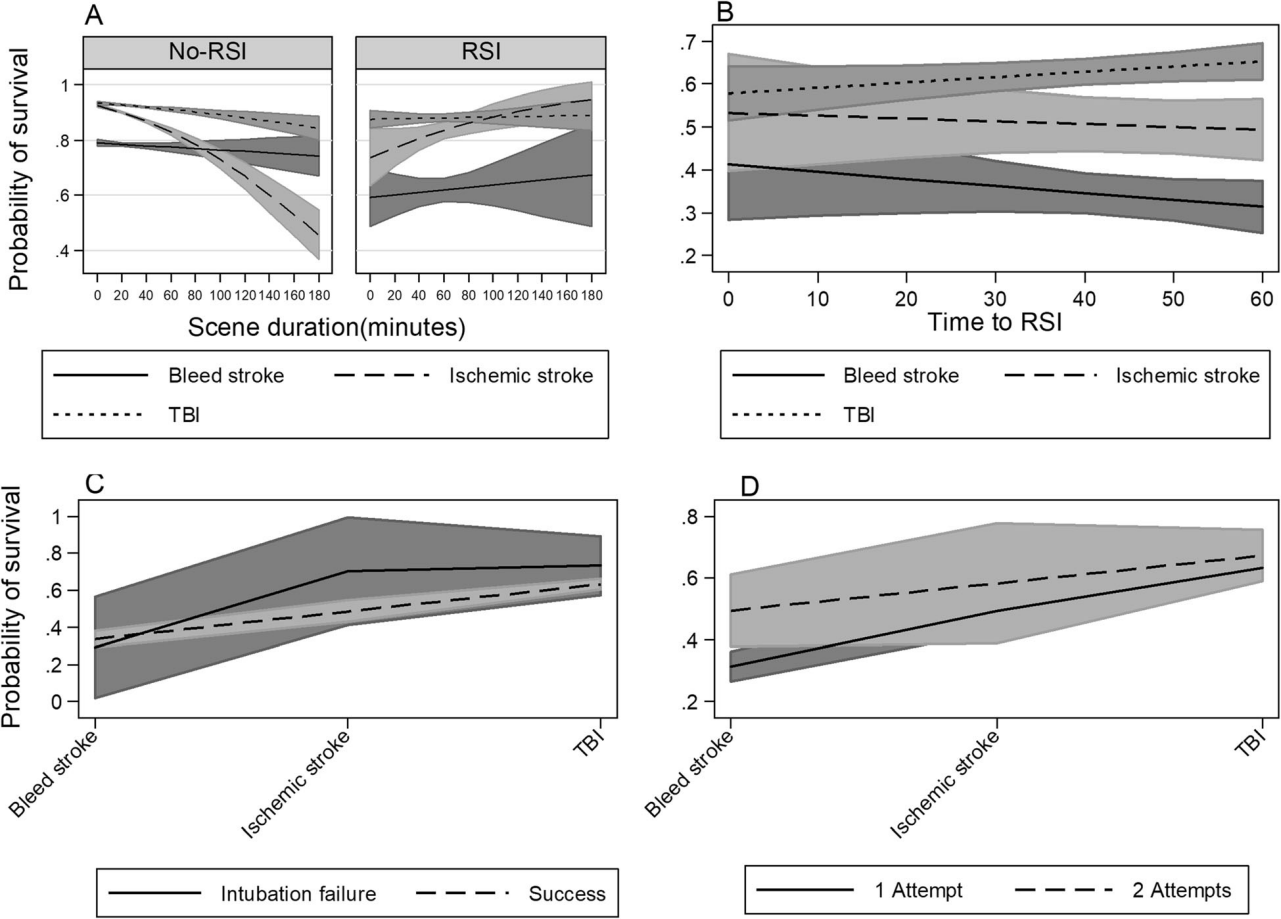
Interactions of time intervals and intubation success in a cohort of strokes and TBI, RSI compared to no-RSI. **a** interaction of scene duration and pathology. **b** interaction of time to RSI and pathology. **c** interaction of intubation success and pathology. **d** interaction of number of intubation attempts and pathology

**Fig. 4:**
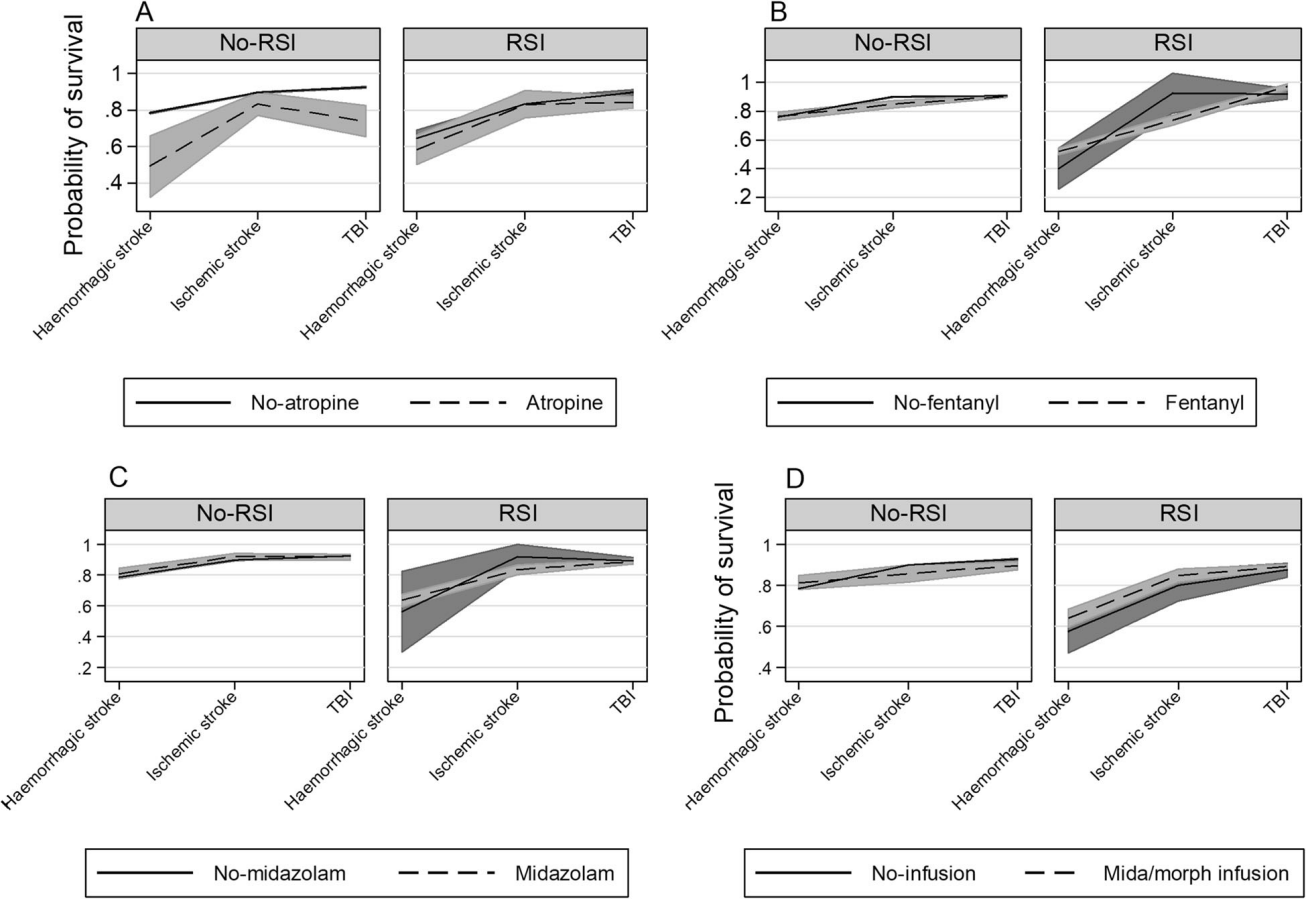
Interactions of medications used with RSI in a cohort of strokes and TBI, RSI compared to no-RS. **a** interaction of atropine and pathology. **b** interaction of fentanyl and pathology. **c** interaction of midazolam and pathology. **d** interaction of midazolam/morphine infusion and pathology

**Fig. 5:**
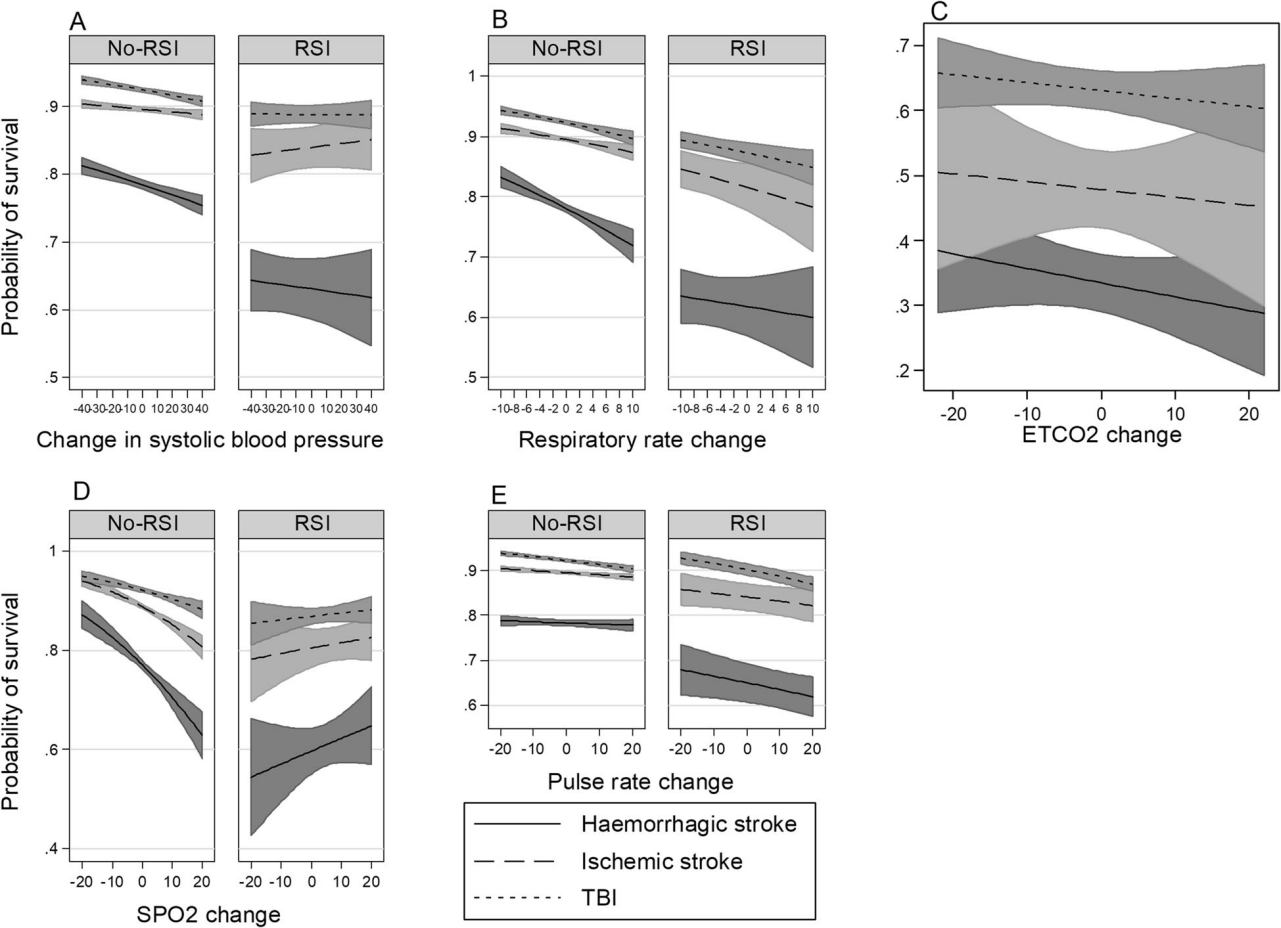
Interactions of changes in vital signs in a cohort of strokes and TBI, RSI compared to no-RSI. **a** change in systolic blood pressure and pathology. **b** interaction of respiratory rate change and pathology. **c** interaction of ETCO2 change and pathology. **d** interaction of SPO2 change and pathology. **e** interaction pulse rate change and pathology

Here we report interactions that are statistically significant or borderline significant. All interactions are presented graphically in Figs. 2, 3, 4 and 5. For a complete report on all interactions, see Additional file 1: Table S2A to O.

### Demographic factors

Age strongly predicted survival (Additional file 1: Table S1A to C). Age interacted with TBI and stroke in the RSI-only group (*p* = 0.02) (Additional file 1: Table S2A; Fig. 2a). A borderline insignificant interaction within the RSI group exist when comparing sex (*p* = 0.06) (Additional file 1: Table S2B; Fig. 2b).

### Prehospital time intervals and intubation success

No significant interaction for scene-time was found in the RSI-only setting (*p* = 0.18) (Additional file 1: Table S2C; Fig. 3a). Even so, there was a borderline significant difference in the slopes for ischemic stroke vs TBI (*p* = 0.06). The mean time-to-RSI was 39.4 min (SD = 24.8), but there was no significant interaction for time-to-RSI (*p* = 0.10) (Additional file 1: Table S2D; Fig. 3b). However, a borderline significant difference between the survival slopes of TBI versus haemorrhagic strokes was noted (*p* = 0.05). We found no evidence of interaction of intubation success (*p* = 0.49) (Additional file 1: Table S2E; Fig. 3c). Two or more attempts at intubation success was associated with increased survival for haemorrhagic strokes (*p* = 0.004), but not for ischemic strokes or TBI (Additional file 1: Table S2F; Fig. 3d). Nonetheless, both the number of intubation attempts and intubation success had low cell counts, limiting inferences from these two analyses.

### Rapid sequence intubation medications

The interaction of atropine showed that survival was lower for TBI when atropine was used with RSI (*p* = 0.001) (Additional file 1: Table S2G; Fig. 4a). The interaction of fentanyl indicated that when fentanyl was used with RSI, survival is better for TBI (*p* = 0.01) (Additional file 1: Table S2H; Fig. 4b). No significant interactions for midazolam was apparent (*p* = 0.34) (Additional file 1: Table S2I; Fig. 4c), nor for midazolam/morphine infusions (*p* = 0.31) and (*p* = 0.84) (Additional file 1: Table S2J; Fig. 4d).

### Changes in vital signs after RSI use

Survival decreased for haemorrhagic strokes and TBI as systolic blood pressure increased (Additional file 1: Table S1A to C). No interaction of systolic blood pressure for the RSI-only group is apparent (*p* = 0.61) (Additional file 1: Table S2K; Fig. 5a). No interaction was found for changes in respiratory rate (*p* = 0.52) (Additional file 1: Table S2L; Fig. 5b). For changes in ETCO_2_ with RSI use, no interaction is obvious (*p* = 0.91) (Additional file 1: Table S2M; Fig. 5c). No interaction for changes in SPO_2_ and TBI and stroke was found in the RSI-only setting (*p* = 0.91) (Additional file 1: Table S2N; Fig. 5d). For those that receive RSI significant differences exist between the stroke and TBI slopes of pulse rate change (*p* = 0.03) (Additional file 1: Table S2O); Fig. 5e).

## Discussion

This analysis shows that RSI interact with strokes and traumatic brain injuries in terms of survival, indicating that the TBI evidence might not be useful to guide stroke RSI. As anticipated, this implies that the stroke patient differs from the TBI patient enough that RSI components impacts survival differently for these two pathologies. Significant interactions in the RSI-only group included number of intubation attempts, atropine, fentanyl, pulse rate and perhaps scene time, time to RSI as well as the age of the patient. Another suggestion that TBI evidence might not transferable is the difference in survival between the strokes and TBI for RSI. Haemorrhagic strokes have 14% lesser RSI survival, compared to ischemic stroke of 4% and TBI a lesser than 1% survival difference between RSI and no-RSI.

Large differences in the changes in systolic blood pressure with RSI use are evident when comparing strokes and TBI. The largest decreases in RSI-related blood pressure were found in haemorrhagic strokes, and the least in TBI. Survival follows this pattern too, with haemorrhagic stroke the poorest survival compared to TBI the best. However, it is not clear that these decreases in blood pressure are the causes of poorer survival. Our analysis show that decreases in blood pressure with RSI was not associated with a significant decrease in survival. This finding of no decrease in survival after a decrease in blood pressure with RSI is mirrored in the results of the GOLIATH trial, which compared conscious sedation to general anaesthesia in ischemic stroke [15]. In the GOLIATH trial general anaesthesia caused a greater mean blood pressure drop compared to the conscious sedation group, similar to the drop in systolic blood pressure found with RSI in our analysis. Despite the decreased blood pressure caused by general anaesthesia in the GOLIATH trial, no decreased survival with such a blood pressure drop was found. Interestingly, survival seems to *increase* for haemorrhagic stroke after decreased blood pressure, but this effect was not statistically significant. This suggestion of increased survival with decreasing blood pressure after clinical interventions in haemorrhagic strokes have been found in other studies [16].

Age could be the most important driver of the differences in RSI survival between strokes and TBI, not blood pressure. Age decreases survival for both strokes and TBI, but the survival decrease is much more rapid for haemorrhagic strokes. These differences in the steepness of decline of the age slopes are important when one considers that almost three quarters of all stroke RSI in our analysis were haemorrhagic strokes, and the average age of these haemorrhagic stokes were 65 years, compared to TBI of 43 years. Given this large difference in age between TBI and haemorrhagic stroke for those that received RSI, and keeping in mind the much steeper decline with age in survival when comparing haemorrhagic stokes to TBI, the large difference between the survival for haemorrhagic stroke compared to TBI is unsurprising. Large decreases in survival with advanced age for patients with haemorrhagic strokes are consistent with another Australian study [17].

Other important interactions in the RSI-only group were evident. An increased number of intubation attempts were associated with increased survival for haemorrhagic strokes. This surprising and possibly confounded effect could result from less obtunded patients needing more intubation attempts. Another significant interaction was atropine, with lower survival for TBI when atropine was used. The mechanisms of this finding are unexplained. Furthermore, the interaction of fentanyl on the pathologies showed TBI have better survival when fentanyl is used and ischemic strokes worse survival with fentanyl use. Fentanyl is used to blunt the hemodynamic effects of intubation [18], but causes a decrease in mean arterial pressure [19]. it is possible that the blood pressure effects of fentanyl could be the cause of this medications impact on survival in this analysis. Furthermore, our analysis also found an interaction between pulse rate and pathology, with both strokes and TBI associated with decreased survival with increased pulse rate. We believe that the effect of pulse rate is mainly through the impact of blood pressure changes, and not pulse rate itself. Also, RSI use is associated with an apparent reversal of the deleterious effect of staying on scene longer. This could be due to potential benefits of RSI or perhaps a selection effect: patient that lived long enough to receive RSI at later scene times would cause an association of better survival with longer times on scene.

It is a strength of our study that our measures of prognostic risk due to illness severity and comorbidities together predicted survival well with an area under curve of 0.83, which is higher than the 0.75 that would provide evidence of adequate risk adjustment [20]. Even so, it is possible that these results are still somewhat biased due to unmeasured confounding. Another strong point of our study is that it had a very large sample size, and consequently good statistical precision. This study has limitations. Firstly, the utility of observational methods to reveal treatment effects are limited, especially if the anticipated effect is small [21]. Secondly, we could not compare good neurological survival, which would have been a more suitable. Thirdly, we calculated the change in vital signs by subtracting the first values upon scene arrival from the last values before handover at the emergency department. Additionally, we believe that comparing the first vital signs measured upon arrival at the patient’s side to the last vital signs (measured after all prehospital treatments were given) would show the impact of the effects of RSI on these vital signs after adjustment for confounders. Our data did not allow for a comparison of vial sign changes *during* RSI. We did not consider non-linear interaction terms due to restrictions in the sample sizes of the RSI group, although we anticipated non-linear effects for many variables.

## Conclusions

Rapid sequence intubation and related factors interact with stroke and TBI, which implies that RSI effects stroke survival differently from TBI. If RSI impact survival differently for strokes compared to TBI, then perhaps the TBI evidence cannot be used for stroke RSI. A trial comparing RSI to no-RSI in the out-of-hospital setting is urgently needed.

## Supplementary information

**Additional file 1: Table S1.** A. Estimates of a logistic regression model of RSI adjusted for covariates in 42,437 traumatic brain injuries. B. Estimates of a logistic regression model of RSI adjusted for covariates in 29,457 ischemic strokes. C. Estimates of a logistic regression model of RSI adjusted for covariates in 14,374 haemorrhagic strokes. **Table S2.** Rapid sequence intubation effect modification of factors. **Table S3.** Model Fit and performance statistics.

## Data Availability

Data is not available due to privacy concerns of patients.

## Abbreviations

GCS: Glasgow Coma Scale
RSI: Rapid Sequence intubation
TBI: Traumatic brain injury
