# Supplementary material for: The utility of the brain trauma evidence to inform paramedic rapid sequence intubation in out-of-hospital stroke
Source: BMC Emerg Med. 2020 Jan 28;20:5. doi: 10.1186/s12873-020-0303-9 (PMC6988411; doi:10.1186/s12873-020-0303-9)
Supplement: Supplementary file 1 — Additional file 1: Table S1. A. Estimates of a logistic regression model of RSI adjusted for covariates in 42,437 traumatic brain injuries. B. Estimates of a logistic regression model of RSI adjusted for covariates in 29,457 ischemic strokes. C. Estimates of a logistic regression model of RSI adjusted for covariates in 14,374 haemorrhagic strokes. Table S2. Rapid sequence intubation effect modification of factors. Table S3. Model Fit and performance statistics. [file 12873_2020_303_MOESM1_ESM.docx]

## Additional file 1

Table S1A Estimates of a logistic regression model of RSI adjusted for covariates in 42,437 traumatic brain injuries

| **Factor** | **Adjusted OR (95% CI)** | **P-value** |
| --- | --- | --- |
| Age (years)∗  40  60  80 | 1.00 (ref)  0.96 (0.97 to 0.97)  0.91 (0.90 to 0.92) | <0.001  <0.001 |
| Elixhauser-Walraven score†  10  20  30  40 | 1.00 (ref)  0.67 (0.62 to 0.72)  0.34 (0.28 to 0.41)  0.14 (0.10 to 0.20) | <0.001  <0.001  <0.001 |
| Initial SPO_2_ (%) ®  100  90  80  70 | 1.00 (ref)  0.57 (0.50 to 0.60)  0.36 (0.28 to 0.48)  0.26 (0.18 to 0.37) | <0.001  <0.001  <0.001 |
| Initial blood sugar level (mmol/l)^μ^  3  7  10  20 | 1.7 (1.5 to 1.9)  1.00 (ref)  0.75 (0.70 to 0.80)  0.37 (0.29 to 0.47) | <0.001  <0.001  <0.001 |
| Glasgow Coma Scale ^χ^  3  6  9  12  15 | 1.00 (ref)  1.69 (1.60 to 1.70)  4.02 (3.68 to 4.39)  13.60 (11.52 to 16.06)  65.15 (49.94 to 85.00) | <0.001  <0.001  <0.001  <0.001 |
| Respiratory rate (per minute) | 0.99 (0.98 to 1.00) | 0.20 |
| Systolic blood pressure (mmHg)^¥^  50  90  120  160 | 1.03 (1.02 to 1.06)  1.00 (ref)  0.94 (0.90 to 0.97)  0.81 (0.73 to 0.91) | <0.001  <0.001  <0.001 |
| Pulse rate(per minute)^◊^  50  100  120  160 | 0.99 (0.99 to 1.00)  1.00 (ref)  1.001 (1.00 to 1.002)  1.001 (1.00 to 1.002) | <0.001  <0.001  <0.001 |
| Sex  Female  Male | 1.00 (ref)  0.72 (0.62 to 0.85 | <0.001 |
| Rapid Sequence Intubation  No-RSI  RSI | 1.00 (ref)  0.86 (0.67 to 1.11) | 0.25 |
| ∗Age fitted as fractional polynomial term *β*1 (age+1/100)^3^; ®Initial SPO_2_ fitted as fractional polynomial term *β*1 (SPO_2_ +1/100)^3^; †Elixhauser fitted as fractional polynomial terms β1 (Elixhauser+1/10)^2^; χ Glasgow Coma Scale fitted as fractional polynomial terms β1 (GCS/10)^2^; ¥ Systolic BP fitted as fractional polynomial term *β*1 (systolic BP+1/100)^3^; ◊Pulse rate fitted as fractional polynomial term *β*1 (pulse+1/100)^-1^ | | |

Table S1B Estimates of a logistic regression model of RSI adjusted for covariates in 29,457 ischemic strokes

| **Factor** | **Adjusted OR (95% CI)** | **P-value** |
| --- | --- | --- |
| Age (years)∗  40  60  80 | 1.00 (ref)  0.97 (0.96 to 0.97)  0.92 (0.91 to 0.93) | <0.001  <0.001 |
| Elixhauser-Walraven score†  10  20  30  40 | 1.00 (ref)  0.84 (0.81 to 0.87)  0.63 (0.57 to 0.69)  0.42 (0.34 to 0.50) | <0.001  <0.001  <0.001 |
| Initial SPO_2_ (%) ®  100  90  80  70 | 1.00 (ref)  0.63 (0.56 to 0.71)  0.43 (0.35 to 0.54)  0.32 (0.24 to 0.43) | <0.001  <0.001  <0.001 |
| Initial blood sugar level (mmol/l) | 0.95 (0.94 to 0.96) | <0.001 |
| Glasgow Coma Scale ^χ^  3  6  9  12  15 | 1.00 (ref)  1.19 (1.18 to 1.20)  1.92 (1.85 to 1.99)  4.86 (4.46 to 5.29)  22.43 (18.96 to 26.53) | <0.001  <0.001  <0.001  <0.001 |
| Respiratory rate (per minute) | 0.96 (0.95 to 0.97) | <0.001 |
| Pulse rate (per minute | 0.99 (0.99 to 1.00) | 0.053 |
| Year | 1.04 (1.02 to 1.07) | <0.001 |
| Rapid Sequence Intubation  No-RSI  RSI | 1.00 (ref)  0.67 (0.49 to 0.91) | 0.01 |
| ∗Age fitted as fractional polynomial term *β*1 (age+1/100)^3^; ® SPO_2_ fitted as fractional polynomial term *β*1 (SPO_2_ +1/100)^3^; †Elixhauser fitted as fractional polynomial terms β1 (Elixhauser/10)^2^; χ Glasgow Coma Scale fitted as fractional polynomial terms β1 (GCS/10)^3^; | | |

Table S1C Estimates of a logistic regression model of RSI adjusted for covariates in 14,374 haemorrhagic strokes

| **Factor** | **Adjusted OR (95% CI)** | **P-value** |
| --- | --- | --- |
| Response duration | 1.001 (1.00 to 1.01) | 0.001 |
| Age (years)∗  40  60  80 | 1.00 (ref)  0.91 (0.90 to 0.92)  0.80 (0.78 to 0.82) | <0.001  <0.001 |
| Elixhauser-Walraven score†  10  20  30  40 | 1.00 (ref)  1.95 (1.68 to 2.27)  2.62 (2.22 to 3.36)  3.13 (2.42 to 4.04) | <0.001  <0.001  <0.001 |
| Initial SPO_2_ (%) ®  100  90  80  70 | 1.00 (ref)  0.49 (0.42 to 0.59)  0.28 (0.21 to 0.39)  0.18 (0.12 to 0.28) | <0.001  <0.001  <0.001 |
| Respiratory rate≈  5  10  20  30 | 1.00 (ref)  1.02 (1.00 to 1.04)  1.03 (1.01 to 1.05)  1.03 (1.01 to 1.05) | 0.01  0.01  0.01 |
| Initial blood sugar level (mmol/l) | 0.96 (0.94 to 0.98) | <0.001 |
| Glasgow Coma Scale ^χ^  3  6  9  12  15 | 1.00 (ref)  1.47 (1.43 to 1.51)  2.80 (2.60 to 3.02)  6.92 (6.01 to 7.95)  22.06 (17.63 to 27.61) | <0.001  <0.001  <0.001  <0.001 |
| Systolic blood pressure (mmHg)^¥^  50  90  120  160 | 1.05 (1.04 to 1.06)  1.00 (ref)  0.92 (0.90 to 0.94)  0.76 (0.71 to 0.82) | <0.001  <0.001  <0.001 |
| Rapid Sequence Intubation  No-RSI  RSI | 1.00 (ref)  0.44 (0.33 to 0.58) | 0.01 |
| ∗Age fitted as fractional polynomial term *β*1 (age+1/100)^2^; ® SPO_2_ fitted as fractional polynomial term *β*1 (SPO_2_ +1/100)^3^; †Elixhauser fitted as fractional polynomial terms β1 (Elixhauser/10) ^-0.5^; χ Glasgow Coma Scale fitted as fractional polynomial terms β1 (GCS/10)^2^; ≈Respiratory rate fitted as fractional polynomial term *β*1 (respiratory rate+1/10)^-2^; ¥ Systolic BP fitted as fractional polynomial term *β*1 (systolic BP+1/100)^3^ | | |

**Table S2 Rapid sequence intubation effect modification of factors**

| **A** | **Age < 58 years** | | **Age ≥ 58 years** | |  |  |
| --- | --- | --- | --- | --- | --- | --- |
| **RSI** | *No. with/without*  *survive to discharge* | *OR (95% CI); P* | *No. with/without*  *survive to discharge* | *OR (95% CI); P* | *OR (95% CI); P for older vs younger age within strata* |  |
| *Haemorrhagic stroke* | 61/102 | **1.0 (REF)** | 57/367 | 0.2 (0.1 – 0.4); p<0.001 | 0.2 (0.1 – 0.4); p<0.001 |  |
| *Ischemic stroke* | 34/33 | 1.1 (0.6 – 2.1);p=0.73 | 74/108 | 0.6 (0.4 – 1.0); p=0.06 | 0.5 (0.3 – 1.0); p=0.06 |  |
| *Traumatic brain injury* | 599/135 | 6.6 (4.3 – 10.1); p<0.001 | 139/157 | 1.3 (0.8 – 2.0); p=0.27 | 0.2 (0.1 – 0.3); p<0.001 |  |
| **No-RSI** | | | | | | |
| *Haemorrhagic stroke* | 2,571/ 423 | **1.0 (REF)** | 5,869/ 3,075 | 0.2 (0.2 – 0.3); p<0.001 | 0.2 (0.2 – 0.3); p<0.001 |  |
| *Ischemic stroke* | 3,107/ 291 | 1.4 (1.2 – 1.8); p=0.001 | 20,750/ 3,662 | 0.6 (0.5 – 0.7); p<0.001 | 0.4 (0.3 – 0.5); p<0.001 |  |
| *Traumatic brain injury* | 33,293/252 | 10.6 (8.6 – 12.9); p<0.001 | 19,666/ 1,662 | 0.8 (0.7 – 0.9); p=0.004 | 0.1 (0.07 – 0.1); p<0.001 |  |
| Estimates are adjusted for GCS, sex, year, respiratory rate, pulse rate, systolic blood pressure and Elixhauser comorbidity score. Fifty eight was the mean age of the sample.  Relative excess risk due to interaction for combined strokes vs. TBI for older vs younger age is -4.5 (-6.6 – -2.3); p<0.001 for RSI and -7.8 (-9.4 - -6.2); p<0.001for no-RSI. | | | | | | |

| **B** | **Female** | | **Male** | |  |  |
| --- | --- | --- | --- | --- | --- | --- |
| **RSI** | *No. with/without*  *survive to discharge* | *OR (95% CI); P* | *No. with/without*  *survive to discharge* | *OR (95% CI); P* | *OR (95% CI); P for male vs female within strata* |  |
| *Haemorrhagic stroke* | 58/259 | **1.0 (REF)** | 60/211 | 1.4 ( 0.9 – 2.3); p = 0.18 | 1.5 (0.9 – 2.5);p=0.12 |  |
| *Ischemic stroke* | 44/67 | 3.2 (1.8- 5.7); p<0.001 | 64/74 | 2.6 (1.5 - 4.4); p=0.001 | 0.9 (0.5 – 1.6);p=0.73 |  |
| *Traumatic brain injury* | 195/73 | 8.7 (5.3 – 14.2); p<0.001 | 544/220 | 6.2 (4.1 – 9.4); p<0.001 | 0.7 (0.5 – 1.0);p=0.07 |  |
| **No-RSI** | | | | | | |
| *Haemorrhagic stroke* | 4,305/ 1,840 | **1.0 (REF)** | 4,135/ 1,659 | 0.9 (0.8 – 1.0); p=0.004 | 0.8 (0.7 – 0.9); p<0.001 |  |
| *Ischemic stroke* | 11,031/ 2,155 | 3.0 (2.7 – 3.3); p<0.001 | 12,831/ 1,801 | 2.9 (2.6 – 3.1); p<0.001 | 1.1 (1.0 – 1.2); p=0.06 |  |
| *Traumatic brain injury* | 19,031/ 799 | 4.9 (4.4 – 5.5); p<0.001 | 33,954/ 1,115 | 4.3 (3.9 – 4.7); p<0.001 | 0.7 (0.7 – 0.8); p<0.001 |  |
| Estimates are adjusted for age, GCS, sex, year, respiratory rate, pulse rate, systolic blood pressure, Elixhauser comorbidity score  Relative excess risk due to interaction for combined strokes vs. TBI for female vs male is 0.07 (-0.04 – 0.18);p=0.23 for RSI and -0.04 (-0.1 - -0.01); p=0.005 for no-RSI | | | | | | |

| **C** | **Shorter scene time (<24 minutes)** | | **Longer scene time (≥24 minutes)** | |  |  |
| --- | --- | --- | --- | --- | --- | --- |
| **RSI** | *No. with/without*  *survive to discharge* | *OR (95% CI); P* | *No. with/without*  *survive to discharge* | *OR (95% CI); P* | *OR (95% CI); P for increased vs decreased scene time within strata* |  |
| *Haemorrhagic stroke* | 9/29 | **1.0 (REF)** | 108/441 | 1.3 (0.5 – 3.3);p=0.63 | 1.3 (0.5 – 3.5);p=0.66 |  |
| *Ischemic stroke* | 9/10 | 4.0 (1.0 – 16.1);p=0.06 | 99/130 | 3.0 (1.1 – 8.1);p=0.03 | 0.8 (0.3 – 2.5);p=0.74 |  |
| *Traumatic brain injury* | 42/14 | 8.6 (2.7 – 27.5); p<0.001 | 684/277 | 7.1 (2.7 – 18.5); p<0.001 | 0.8 (0.4 – 1.6);p=0.54 |  |
| **No-RSI** | | | | | | |
| *Haemorrhagic stroke* | 5,478 / 1,670 | **1.0 (REF)** | 2,919 /1,815 | 0.8 (0.8 – 0.9); p<0.001 | 0.8 (0.7 – 0.9); p<0.001 |  |
| *Ischemic stroke* | 17,225 / 2,094 | 3.5 (3.2 – 3.8); p<0.001 | 6,584 /1,849 | 2.2 (2.0 – 2.4); p<0.001 | 0.7 (0.6 – 0.7); p<0.001 |  |
| *Traumatic brain injury* | 32,754 /814 | 5.0 (4.5 – 5.6); p<0.001 | 20,030 /1,094 | 4.0 (3.7 – 4.5); p<0.001 | 0.8 (0.8 – 0.9); p=0.003 |  |
| Estimates are adjusted for age, GCS, sex, year, respiratory rate, pulse rate, systolic blood pressure and Elixhauser comorbidity score. 24 minutes was the mean scene time  Relative excess risk due to interaction for combined strokes vs. TBI for unchanged/increased shorter versus longer scene times  is -0.8 (-4.5 – 2.9); p=0.66 for RSI and -0.07(-0.3 - 0.2); p=0.53 for no-RSI. | | | | | | |

| **D** | **Shorter time to RSI(<39 minutes)** | | **Longer time to RSI(≥39 minutes)** | |  |  |
| --- | --- | --- | --- | --- | --- | --- |
| **RSI** | *No. with/without*  *survive to discharge* | *OR (95% CI); P* | *No. with/without*  *survive to discharge* | *OR (95% CI); P* | *OR (95% CI); P for shorter vs longer time-to-RSI within strata* |  |
| *Haemorrhagic stroke* | 44/174 | **1.0 (REF)** | 59/235 | 0.7 (0.4 – 1.2);p=0.19 | 0.8 (0.4 – 1.4);p=0.37 |  |
| *Ischemic stroke* | 41/52 | 2.5 (1.3 – 4.7);p=0.01 | 55/69 | 1.9 (1.0 – 3.4);p=0.04 | 0.7 (0.4 – 1.5);p=0.37 |  |
| *Traumatic brain injury* | 308/152 | 3.9 (2.5 – 6.3); p<0.001 | 316/102 | 5.1 (3.1 – 8.3); p<0.001 | 1.3 (0.9 – 1.9);p=0.22 |  |
| Estimates are adjusted for age, GCS, sex, year, respiratory rate, pulse rate, systolic blood pressure and Elixhauser comorbidity score. 39 minutes is the mean time to RSI.  Relative excess risk due to interaction for combined strokes vs. TBI for midazolam vs none = 1.09 (-0.1 – 2.3);p=0.08 for RSI | | | | | | |

| **E** | **Intubation failure** | | **Intubation success** | |  |  |
| --- | --- | --- | --- | --- | --- | --- |
| **RSI** | *No. with/without*  *survive to discharge* | *OR (95% CI); P* | *No. with/without*  *survive to discharge* | *OR (95% CI); P* | *OR (95% CI); P for success vs failure within strata* |  |
| *Haemorrhagic stroke* | 3/14 | **1.0 (REF)** | 115/455 | 1.3 (0.2 – 7.6); p=0.76 | 1.5 (0.2 – 8.8);p=0.67 |  |
| *Ischemic stroke* | 3/2 | 11.4 (0.8 – 157.2); p=0.07 | 105/138 | 3.1 (0.5 – 18.3); p=0.21 | 0.3 (0.04 – 2.2);p=0.23 |  |
| *Traumatic brain injury* | 15/6 | 14.1 (1.8 – 113.2); p=0.01 | 719/287 | 7.3 (1.3 – 41.8); p=0.03 | 0.5 (0.1 – 1.6);p=0.22 |  |
| Estimates are adjusted for age, GCS, sex, year, respiratory rate, pulse rate, systolic blood pressure, Elixhauser comorbidity score  Relative excess risk due to interaction for combined strokes vs. TBI for intubation success vs failure = -2.3 (-9.6 – 5.0); p=0.54 | | | | | | |

| **F** | **Intubation attempts (one)** | | **Intubation attempts (two or more)** | |  |  |
| --- | --- | --- | --- | --- | --- | --- |
| **RSI** | *No. with/without*  *survive to discharge* | *OR (95% CI); P* | *No. with/without*  *survive to discharge* | *OR (95% CI); P* | *OR (95% CI); P for one vs two or more intubation attempts within strata* |  |
| *Haemorrhagic stroke* | 87/379 | **1.0 (REF)** | 18/40 | 2.9 (1.4 – 6.0); p=0.004 | 2.9 (1.4 – 6.3);p=0.01 |  |
| *Ischemic stroke* | 86/109 | 2.9 (1.8 – 4.2); p<0.001 | 10/9 | 4.8 (1.5 – 15.9); p=0.01 | 1.6 (0.5 – 4.8);p=0.43 |  |
| *Traumatic brain injury* | 594/239 | 6.6 (4.6 – 9.4); p<0.001 | 71/23 | 8.4 (4.5 – 15.8); p<0.001 | 1.2 (0.7 – 2.3);p=0.47 |  |
| Estimates are adjusted for age, GCS, sex, year, respiratory rate, pulse rate, systolic blood pressure, Elixhauser comorbidity score  Relative excess risk due to interaction for combined strokes vs. TBI for one attempt vs two or more = 0.92 (0.5 – 1.3); p<0.001 | | | | | | |

| **G** | **No- atropine** | | **Atropine** | |  |  |
| --- | --- | --- | --- | --- | --- | --- |
| **RSI** | *No. with/without*  *survive to discharge* | *OR (95% CI); P* | *No. with/without*  *survive to discharge* | *OR (95% CI); P* | *OR (95% CI); P for atropine vs No-atropine within strata* |  |
| *Haemorrhagic stroke* | 87/353 | 1.0 (REF) | 31/117 | 0. 8 ( 0.5 - 1.4); p = 0.40 | 1.1 (0.6 – 1.9);p=0.86 |  |
| *Ischemic stroke* | 91/119 | 2.3 (1.5- 3.6); p<0.001 | 17/22 | 2.3 (1.1 - 5.1); p=0.04 | 0.9 (0.4 – 2.1);p=0.90 |  |
| *Traumatic brain injury* | 585/214 | 6.1 (4.3 - 8.7); p<0.001 | 154/79 | 3.5 (2.2 – 5.4); p<0.001 | 0.5 (0.4 – 0.7);p=0.001 |  |
| **No-RSI** | | | | | |  |
| *Haemorrhagic stroke* | 8,432/3,460 | 1.0 (REF) | 8/39 | 0.2 (0.07 - 0.4); p<0.001 | 0.2 (0.07 – 0.4); p<0.001 |  |
| *Ischemic stroke* | 23,826/3,930 | 3.1 (2. – 3.3); p<0.001 | 36/26 | 1.6 (0.9 – 3.0); p=0.13 | 0.7 (0.4 – 1.3); p=0.25 |  |
| *Traumatic brain injury* | 52,957/1,881 | 4.9 (4.6 – 5.3); p<0.001 | 28/33 | 0.7 (0.4 – 1.3); p=0.29 | 0.1 (0.07 – 0.3); p<0.001 |  |
| Estimates are adjusted for age, GCS, sex, year, respiratory rate, pulse rate, systolic blood pressure, Elixhauser comorbidity score  Relative excess risk due to interaction for combined strokes vs. TBI for atropine vs. none is -0.06 (-0.2 – 0.05); p=0.31 for RSI and -1.4 (-1.7 – -1.1); p<0.001 for no-RSI. | | | | | | |

| **H** | **No- fentanyl** | | **Fentanyl** | |  |  |
| --- | --- | --- | --- | --- | --- | --- |
| **RSI** | *No. with/without*  *survive to discharge* | *OR (95% CI); P* | *No. with/without*  *survive to discharge* | *OR (95% CI); P* | *OR (95% CI); P for fentanyl vs. no-fentanyl within strata* |  |
| *Haemorrhagic stroke* | 2/16 | **1.0 (REF)** | 116/454 | 4.2 ( 0.5 – 34.8); p = 0.18 | 5.9 (0.7 – 50.2);p=0.10 |  |
| *Ischemic stroke* | 10/7 | 11.7 (1.1- 130.1); p=0.045 | 98/134 | 9.7 (1.2 - 80.9); p=0.04 | 0.7 (0.2 – 2.3);p=0.54 |  |
| *Traumatic brain injury* | 175/75 | 15.9 (1.9 – 132.8); p=0.01 | 564/218 | 25.8 (3.1 – 212.3); p=0.003 | 1.7 (1.1 – 2.6);p=0.02 |  |
| **No-RSI** | | | | | | |
| *Haemorrhagic stroke* | 8,148/3,399 | **1.0 (REF)** | 292/100 | 0.8 (0.6 – 1.1); p=0.12 | 0.8 (0.6 – 1.1); p=0.24 |  |
| *Ischemic stroke* | 23,519/3,870 | 3.1 (2.9 – 3.4); p<0.001 | 343/86 | 1.9 (1.5 – 2.6); p<0.001 | 0.7 (0.5 – 0.9); p=0.006 |  |
| *Traumatic brain injury* | 48,381/1,804 | 4.7 (4.4 – 5.1); p<0.001 | 4,604/110 | 6.2 (5.0 – 7.7); p<0.001 | 1.3 (0.99 – 1.6); p=0.05 |  |
| Estimates are adjusted for age, GCS, sex, year, respiratory rate, pulse rate, systolic blood pressure, Elixhauser comorbidity score  Relative excess risk due to interaction for combined strokes vs. TBI for fentanyl vs none is 0.17 (0.03 – 0.30); p=0.01 for RSI and -0.06 (-0.1 - 0.0002); p=0.05for no-RSI. | | | | | | |

| **I** | **No- midazolam** | | **Midazolam** | |  |  |
| --- | --- | --- | --- | --- | --- | --- |
| **RSI** | *No. with/without*  *survive to discharge* | *OR (95% CI); P* | *No. with/without*  *survive to discharge* | *OR (95% CI); P* | *OR (95% CI); P for midazolam vs no-midazolam within strata* |  |
| *Haemorrhagic stroke* | 4/17 | **1.0 (REF)** | 114/453 | 1.8 ( 0.4 – 7.2); p = 0.42 | 2.2 (0.5 – 9.7);p=0.30 |  |
| *Ischemic stroke* | 8/4 | 4.6 (0.6- 33.1); p=0.13 | 100/137 | 4.2 (1.0 - 17.1); p=0.045 | 0.7 (0.2 – 2.9);p=0.61 |  |
| *Traumatic brain injury* | 256/74 | 9.9 (2.5 – 40.1); p=0.001 | 483/219 | 10.0 (2.5 – 40.0); p=0.001 | 0.9 (0.6 – 1.5);p=0.79 |  |
| **No-RSI** | | | | | | |
| *Haemorrhagic stroke* | 8,321/3,354 | **1.0 (REF)** | 119/145 | 1.3 (0.9 – 1.7); p=0.15 | 1.1 (0.8 – 1.5); p=0.59 |  |
| *Ischemic stroke* | 23,747/3,882 | 3.1 (2.9 – 3.4); p<0.001 | 115/74 | 4.9 (3.3 – 7.1); p<0.001 | 2.0 (1.4 – 3.0); p<0.001 |  |
| *Traumatic brain injury* | 52,709/1,844 | 4.9 (4.6 – 5.3); p<0.001 | 276/70 | 4.5 (3.2 – 6.3); p<0.001 | 0.9 (0.7 – 1.4); p=0.76 |  |
| Estimates are adjusted for age, GCS, sex, year, respiratory rate, pulse rate, systolic blood pressure, Elixhauser comorbidity score  Relative excess risk due to interaction for combined strokes vs. TBI for midazolam vs none = -0.25 (-2.7 – 2.2);p=0.84 for RSI and -0.08 ( -0.9 - 0.8); p=0.85 for no-RSI | | | | | | |

| **J** | **No midazolam/morphine infusion** | | **Midazolam/morphine infusion** | |  |  |
| --- | --- | --- | --- | --- | --- | --- |
| **RSI** | *No. with/without*  *survive to discharge* | *OR (95% CI); P* | *No. with/without*  *survive to discharge* | *OR (95% CI); P* | *OR (95% CI); P for infusion vs no-infusion within strata* |  |
| *Haemorrhagic stroke* | 19/89 | **1.0 (REF)** | 99/381 | 1.3 ( 0.6 – 2.4); p = 0.51 | 1.4 (0.7 – 2.8);p=0.41 |  |
| *Ischemic stroke* | 18/33 | 2.0 (0.8- 4.9); p=0.15 | 90/108 | 3.2 (1.6 - 6.5); p=0.001 | 1.5 (0.7 – 3.3);p=0.34 |  |
| *Traumatic brain injury* | 98/54 | 6.2 (3.0 – 12.8); p<0.001 | 641/239 | 7.1 (3.7 – 13.6); p<0.001 | 1.1 (0.7 – 1.9);p=0.57 |  |
| **No-RSI** | | | | | | |
| *Haemorrhagic stroke* | 8,347/ 3,351 | **1.0 (REF)** | 93/148 | 1.3 (1.0 – 1.8); p=0.09 | 1.1 (0.8 – 1.5); p=0.73 |  |
| *Ischemic stroke* | 23,815/ 3,881 | 3.2 (3.0 – 3.4); p<0.001 | 47/75 | 2.0 (1.3 – 3.2); p=0.002 | 1.1 (0.7 – 1.8); p=0.64 |  |
| *Traumatic brain injury* | 52,784/ 1,810 | 5.0 (4.6 – 5.4); p<0.001 | 201/104 | 3.3 (2.4 – 4.3); p<0.001 | 0.6 (0.4 – 0.8); p=0.001 |  |
| Estimates are adjusted for age, GCS, sex, year, respiratory rate, pulse rate, systolic blood pressure, Elixhauser comorbidity score  Relative excess risk due to interaction for combined strokes vs. TBI for infusion vs none = 0.3 (-1.9 – 2.6);p=0.77 for RSI and -0.4 ( -0.9 - 0.2); p=0.20 for no-RSI | | | | | | |

| **K** | **Unchanged systolic blood pressure** | | **Decreased systolic blood pressure** | | **Increased systolic blood pressure** | | |  |  |  |
| --- | --- | --- | --- | --- | --- | --- | --- | --- | --- | --- |
| **RSI** | *No. with/without*  *survive to discharge* | *OR (95% CI); P* | *No. with/without*  *survive to discharge* | *OR (95% CI); P* | *No. with/without*  *survive to discharge* | | *OR (95% CI); P* | *OR (95% CI); p for BP change within strata (unchanged BP as reference)* |  | |
| *Haemorrhagic stroke* | 6/23 | **1.0 (REF)** | 76/313 | 1.1 (0.3 – 3.7);p=0.91 | 35/129 | | 0.7 (0.2 – 2.5);p=0.60 | Decreased BP: 1.0 (0.3 – 3.7);p=0.96  Increased BP: 0.7 (0.2 – 2.5);p=0.55 |  |  |
| *Ischemic stroke* | 5/8 | 1.9 (0.3 – 11.0);p=0.47 | 57/90 | 2.0 (0.6 – 6.9);p=0.29 | 41/41 | | 2.8 (0.8 – 10.1);p=0.13 | Decreased BP: 0.9 (0.2 – 3.2);p=0.84  Increased BP: 1.3 (0.3 – 5.0);p=0.70 |  |  |
| *Traumatic brain injury* | 45/10 | 7.3 (1.8 – 29.5);p-0.01 | 344/164 | 5.4 (1.6 – 17.9);p=0.01 | 341/116 | | 5.0 (1.5 – 17.0);p=0.01 | Decreased BP: 0.7 (0.3 – 1.6);p=0.44  Increased BP: 0.7 (0.3 – 1.6);p=0.41 |  |  |
| **No-RSI** | | | | | | | | | | |
| *Haemorrhagic stroke* | 2,488/873 | **1.0 (REF)** | 2,989/1,212 | 1.0 (0.9 – 1.2);p=0.46 | 2,085/1,114 | 0.8 (0.7 – 0.9);p=0.001 | | Decreased BP: 1.1 (1.0 – 1.3);p=0.11  Increased BP: 0.8 (0.7 – 0.9); p<0.001 | |  |
| *Ischemic stroke* | 7,628/1,227 | 2.9 (2.6 – 3.2);p<0.001 | 8,473/1,288 | 3.2 (2.9 – 3.6); p<0.001 | 6,565/1,161 | 2.9 (2.6 – 3.3); p<0.001 | | Decreased BP: 1.0 (0.9 – 1.1);p=0.44  Increased BP: 1.1 (1.0 – 1.2);p=0.15 | |  |
| *Traumatic brain injury* | 15,944/493 | 4.5 (4.0 – 5.2) p<0.001 | 19,156/570 | 5.5 (4.8 – 6.3); p<0.001 | 12,956/659 | 3.9 (3.4 – 4.4); p<0.001 | | Decreased BP: 1.3 (1.1 – 1.5);p=0.002  Increased BP: 0.8 (0.7 – 1.0);p=0.01 | |  |
| Estimates are adjusted for age, GCS, sex, year, respiratory rate, pulse rate, systolic blood pressure, and Elixhauser comorbidity score.  Relative excess risk due to interaction for combined strokes vs. TBI for unchanged/increased systolic BP vs decreased = -0.08 (-1.4 – 1.3); p=0.90 for RSI and -0.6(-0.9 - -0.3); p=0.001 for no-RSI. | | | | | | | | | | |

| **L** | **Unchanged respiratory rate** | | | **Decreased**  **respiratory rate** | | **Increased respiratory rate** | | |  |  |  |
| --- | --- | --- | --- | --- | --- | --- | --- | --- | --- | --- | --- |
| **RSI** | *No. with/without*  *survive to discharge* | *OR (95% CI); P* | | *No. with/without*  *survive to discharge* | *OR (95% CI); P* | *No. with/without*  *survive to discharge* | | *OR (95% CI); P* | *OR (95% CI); p for RR change within strata (unchanged RR as reference)* |  | |
| *Haemorrhagic stroke* | 19/78 | **1.0 (REF)** | | 77/291 | 0.8 (0.4 – 1.7);p=0.63 | 20/94 | | 0.8 (0.4 – 1.8);p=0.60 | Decreased RR: 0.8 (0.4 – 1.6);p=0.56  Increased RR: 0.7 (0.3 – 1.6);p=0.41 |  |  |
| *Ischemic stroke* | 19/29 | 2.4 (1.0 – 5.8);p=0.06 | | 72/84 | 2.2 (1.1 – 4.5);p=0.03 | 12/26 | | 1.5 (0.6 – 4.0);p=0.43 | Decreased RR: 1.2 (0.6 – 2.6);p=0.60  Increased RR: 0.5 (0.2 – 1.4);p=0.18 |  |  |
| *Traumatic brain injury* | 128/47 | 5.2 (2.5 – 10.7);p<0.001 | | 486/177 | 5.4 (2.8 – 10.3);p<0.001 | 109/65 | | 3.5 (1.7 – 7.0);p=0.001 | Decreased RR: 1.0 (0.6 – 1.6);p=0.91  Increased RR: 0.7 (0.4 – 1.2);p=0.20 |  |  |
| **No-RSI** | | | | | | | | | | | |
| *Haemorrhagic stroke* | 5,803/2,117 | | **1.0 (REF)** | 1,297/672 | 1.0 (0.9 – 1.1);p=0.96 | 382/410 | 0.6 (0.5 – 0.7); p<0.001 | | Decreased RR: 1.0 (0.9 – 1.2);p=0.74  Increased RR: 0.6 (0.5 – 0.7); p<0.001 | |  |
| *Ischemic stroke* | 17,871/2,579 | | 3.2 (2.9 – 3.4);p<0.001 | 3,566/759 | 3.1 (2.7 – 3.4); p<0.001 | 1,052/326 | 2.4 (2.0 – 2.7); p<0.001 | | Decreased RR: 1.1 (0.9 – 1.2);p=0.35  Increased RR: 0.8 (0.7 – 0.9);p=0.008 | |  |
| *Traumatic brain injury* | 35,135/1,130 | | 4.7 (3.3 – 5.2) p<0.001 | 11,831/386 | 5.4 (4.8 – 6.2); p<0.001 | 2,185/218 | 3.2 (2.7 – 3.9); p<0.001 | | Decreased RR: 1.0 (0.9 – 1.1);p=0.90  Increased RR: 0.6 (0.5 – 0.8); p<0.001 | |  |
| Estimates are adjusted for age, GCS, sex, year, respiratory rate, pulse rate, systolic blood pressure, and Elixhauser comorbidity score. RR=respiratory rate  Relative excess risk due to interaction for combined strokes vs. TBI for unchanged/increased respiratory rate vs decreased is -0.99 (-2.5 – 0.5); p=0.19 for RSI and -0.5(-0.9 – -0.1); p=0.008 for no-RSI. | | | | | | | | | | | |

| **M** | **Unchanged ETCO_2_** | | **Decreased ETCO_2_** | | **Increased ETCO_2_** | |  |  |
| --- | --- | --- | --- | --- | --- | --- | --- | --- |
| **RSI** | *No. with/without*  *survive to discharge* | *OR (95% CI); P* | *No. with/without*  *survive to discharge* | *OR (95% CI); P* | *No. with/without*  *survive to discharge* | *OR (95% CI); P* | *OR (95% CI); p for ETCO_2_ change within strata (unchanged*  *ETCO2 as reference)* |  |
| *Haemorrhagic stroke* | 15/42 | **1.0 (REF)** | 57/238 | 0.6 (0.3 – 1.3);p=0.21 | 45/171 | 0.5 (0.2 – 1.2);p=0.11 | Decreased BP: 0.7 (0.3 – 1.4);p=0.31  Increased BP: 0.6 (0.2 – 1.3);p=0.16 |  |
| *Ischemic stroke* | 12/14 | 2.0 (0.6 – 6.7);p=0.25 | 48/66 | 1.2 (0.5 – 2.8);p=0.66 | 42/57 | 1.4 (0.6 – 3.3);p=0.40 | Decreased BP: 0.7 (0.3 – 2.0);p=0.51  Increased BP: 0.8 (0.3 – 2.2);p=0.62 |  |
| *Traumatic brain injury* | 59/18 | 5.7 (2.2 – 14.4);p-0.01 | 424/159 | 3.3 (1.6 – 6.9);p=0.001 | 218/106 | 3.0 (1.4 – 6.4);p=0.004 | Decreased BP: 0.6 (0.3 – 1.1);p=0.11  Increased BP: 0.5 (0.3 – 1.1);p=0.08 |  |
| Estimates are adjusted for age, GCS, sex, year, respiratory rate, pulse rate, systolic blood pressure, and Elixhauser comorbidity score.  Relative excess risk due to interaction for combined strokes vs. TBI for unchanged/increased ETCO_2_ vs decreased = -0.6 (-2.2 – 0.9);p=0.43 for RSI | | | | | | | | |

| **N** | **Unchanged SPO_2_** | | **Decreased**   **SPO_2_** | | **Increased SPO_2_** | |  |  |  |
| --- | --- | --- | --- | --- | --- | --- | --- | --- | --- |
| **RSI** | *No. with/without*  *survive to discharge* | *OR (95% CI); P* | *No. with/without*  *survive to discharge* | *OR (95% CI); P* | *No. with/without*  *survive to discharge* | *OR (95% CI); P* | *OR (95% CI); p for change of SPO2 within strata (unchanged SPO2 as reference)* |  | |
| *Haemorrhagic stroke* | 32/113 | **1.0 (REF)** | 15/46 | 1.2 (0.5 – 2.9);p=0.64 | 69/307 | 0.9 (0.5 – 1.6);p=0.81 | Decreased: 0.9 (0.4 – 2.4);p=0.90  Increased: 0.9 (0.5 – 1.6);p=0.62 |  |  |
| *Ischemic stroke* | 15/29 | 1.8 (0.8 – 4.2);p=0.19 | 9/9 | 3.1 (0.9 – 10.4);p=0.07 | 78/98 | 2.5 (1.4 – 4.5);p=0.003 | Decreased: 1.9 (0.5 – 6.9);p=0.31  Increased: 1.3 (0.6 – 2.9);p=0.47 |  |  |
| *Traumatic brain injury* | 219/63 | 8.0 (4.5 – 14.3);p=0.001 | 39/34 | 2.7 (1.4 – 5.5);p=0.005 | 469/191 | 5.5 (3.3 – 9.3); p<0.001 | Decreased: 0.3 (0.2 – 0.6);p=0.001  Increased: 0.7 (0.5 – 1.0);p=0.06 |  |  |
| **No-RSI** | | | | | | | | | |
| *Haemorrhagic stroke* | 2,288/ 818 | **1.0 (REF)** | 650/271 | 0.8 (0.7 – 1.0);p=0.03 | 1,248/ 1,083 | 0.7 (0.6 – 0.8); p<0.001 | Decreased: 0.8 (0.7 – 1.0);p=0.05  Increased: 0.6 (0.6 – 0.7); p<0.001 | |  |
| *Ischemic stroke* | 7,231/917 | 3.1 (2.7 – 3.5);p<0.001 | 2,060/272 | 3.0 (2.5 – 3.5); p<0.001 | 3,817/ 1,057 | 2.0 (1.8 – 2.3); p<0.001 | Decreased : 1.0 (0.8 – 1.2);p=0.90  Increased : 0.7 (0.6 – 0.8); p<0.001 | |  |
| *Traumatic brain injury* | 15,581/ 471 | 5.0 (4.3 – 5.7) p<0.001 | 4,491/ 124 | 5.2 (4.2 – 6.5); p<0.001 | 7,850/535 | 3.3 (2.9 – 3.8); p<0.001 | Decreased : 1.1 (0.8 – 1.3);p=0.58  Increased : 0.7 (0.6 – 0.8); <0.001 | |  |
| Estimates are adjusted for age, GCS, sex, year, respiratory rate, pulse rate, systolic blood pressure, and Elixhauser comorbidity score. RR=respiratory rate  Relative excess risk due to interaction for combined strokes vs. TBI for unchanged/increased SPO_2_ vs decreased is 2.3 (0.9 – 3.7); p=0.001 for RSI and -0.4(-1.1 - 0.1); p=0.15 for no-RSI. | | | | | | | | | |

| **O** | **Unchanged pulse rate** | | **Decreased**  **pulse rate** | | **Increased pulse rate** | |  |  |  |
| --- | --- | --- | --- | --- | --- | --- | --- | --- | --- |
| **RSI** | *No. with/without*  *survive to discharge* | *OR (95% CI); P* | *No. with/without*  *survive to discharge* | *OR (95% CI); P* | *No. with/without*  *survive to discharge* | *OR (95% CI); P* | *OR (95% CI); p for*  *pulse rate within strata (unchanged pulse rate as reference)* |  | |
| *Haemorrhagic stroke* | 4/24 | 1.0 (REF) | 38/132 | 1.4 (0.4 – 5.3);p=0.60 | 75/309 | 1.0 (0.3 – 3.5);p=0.99 | Decreased: 1.2 (0.3 – 4.7);p=0.75  Increased: 1.2 (0.3 – 4.4);p=0.81 |  |  |
| *Ischemic stroke* | 11/11 | 3.4 (0.7 – 16.0);p=0.12 | 47/55 | 3.4 (0.9 – 12.8);p=0.07 | 45/73 | 2.0 (0.5 – 7.6);p=0.29 | Decreased: 1.0 (0.3 – 2.7);p=0.95  Increased: 0.5 (0.2 – 1.3);p=0.15 |  |  |
| *Traumatic brain injury* | 36/6 | 15.9 (3.3 – 76.5);p=0.001 | 309/90 | 9.6 (2.7 – 34.1);p<0.001 | 384/194 | 4.7 (1.3 – 16.6);p=0.02 | Decreased: 0.6 (0.2 – 1.7);p=0.36  Increased: 0.3 (0.1 – 0.8);p=0.02 |  |  |
| **No-RSI** | | | | | | | | | |
| *Haemorrhagic stroke* | 2,398/ 807 | 1.0 (REF) | 3,336/1,407 | 0.9 (0.8 – 1.0);p=0.03 | 1,865/1,013 | 0.8 (0.7 – 0.9); p=0.001 | Decreased: 0.9 (0.8 – 1.0);p=0.03  Increased: 0.8 (0.7 – 0.9); p<0.001 | |  |
| *Ischemic stroke* | 6,976/1,045 | 2.8 (2.5 – 3.2);p<0.001 | 10,004/1,478 | 3.0 (2.7 – 3.4); p<0.001 | 5,772/1,178 | 2.5 (2.3 – 2.9); p<0.001 | Decreased : 1.1 (1.0 – 1.2);p=0.19  Increased : 0.9 (0.8 – 1.0);p=0.04 | |  |
| *Traumatic brain injury* | 12,958/ 457 | 4.1 (3.5 – 4.7) p<0.001 | 26,958/ 737 | 5.1 (4.5 – 5.8); p<0.001 | 9,793/554 | 3.5 (3.1 – 4.0); p<0.001 | Decreased : 1.2 (1.0 – 1.3);p=0.04  Increased : 0.8 (0.7 – 1.0); p=0.03 | |  |
| Estimates are adjusted for age, GCS, sex, year, respiratory rate, pulse rate, systolic blood pressure, and Elixhauser comorbidity score. RR=respiratory rate  Relative excess risk due to interaction for combined strokes vs. TBI for unchanged/increased pulse rate vs decreased is -1.8 (-3.4 – 0.2); p=0.03 for RSI and -0.7(-1.0 – -0.5); p<0.001 for no-RSI. | | | | | | | | | |

**Table S3 model Fit and performance statistics**

| Model | No. observations model fitted to | Hosmer and Lemeshow statistic (chi^2^(DF); P) | Pseudo R^2^ | Area under curve | Bayesian Information Criterion |
| --- | --- | --- | --- | --- | --- |
| Baseline model for interactions | 107,128 | 73.7(8); p<0.001 | 0.26 | 0.85 | 22823.1 |
| Baseline model for interactions, random sample* | 4,000 | 11.8(8);p=0.16 | 0.24 | 0.87 | 2146.3 |
| Traumatic brain injury | 63,297 | 39.7(8);p<0.001 | 0.35 | 0.91 | 5118.9 |
| Traumatic brain injury, random sample* | 4,000 | 5.8(8);p=0.67 | 0.33 | 0.89 | 456.9 |
| Haemorrhagic Stroke | 14,374 | 4.1(8);p=0.85 | 0.28 | 0.83 | 5635.5 |
| Haemorrhagic Stroke, random sample* | 4,000 | 10.8(8);p=0.21 | 0.28 | 0.83 | 1638.3 |
| Ischemic stroke | 29,457 | 33.6(8);p<0.001 | 0.22 | 0.83 | 10114.4 |
| Ischemic stroke, random sample | 4,000 | 12.1(8);p=0.15 | 0.24 | 0.83 | 1470.8 |

*Random sample of 4,000 observations
